# CD44 modulates metabolic pathways and altered ROS-mediated Akt signal promoting cholangiocarcinoma progression

**DOI:** 10.1371/journal.pone.0245871

**Published:** 2021-03-29

**Authors:** Malinee Thanee, Hasaya Dokduang, Yingpinyapat Kittirat, Jutarop Phetcharaburanin, Poramate Klanrit, Attapol Titapun, Nisana Namwat, Narong Khuntikeo, Arporn Wangwiwatsin, Hideyuki Saya, Watcharin Loilome

**Affiliations:** 1 Cholangiocarcinoma Screening and Care Program (CASCAP), Khon Kaen University, Khon Kaen, Thailand; 2 Cholangiocarcinoma Research Institute, Khon Kaen University, Khon Kaen, Thailand; 3 Department of Pathology, Faculty of Medicine, Khon Kaen University, Khon Kaen, Thailand; 4 Department of Biochemistry, Faculty of Medicine, Khon Kaen University, Khon Kaen, Thailand; 5 Department of Surgery, Faculty of Medicine, Khon Kaen University, Khon Kaen, Thailand; 6 Division of Gene Regulation, Institute for Advanced Medical Research (IAMR), Keio University School of Medicine, Tokyo, Japan; Duke University School of Medicine, UNITED STATES

## Abstract

CD44 is a transmembrane glycoprotein, the phosphorylation of which can directly trigger intracellular signaling, particularly Akt protein, for supporting cell growth, motility and invasion. This study examined the role of CD44 on the progression of Cholangiocarcinoma (CCA) using metabolic profiling to investigate the molecular mechanisms involved in the Akt signaling pathway. Our results show that the silencing of CD44 decreases Akt and mTOR phosphorylation resulting in p21 and Bax accumulation and Bcl-2 suppression that reduces cell proliferation. Moreover, an inhibition of cell migration and invasion regulated by CD44. Similarly, the silencing of CD44 showed an alteration in the epithelial-mesenchymal transition (EMT), e.g. an upregulation of E-cadherin and a downregulation of vimentin, and the reduction of the matrix metalloproteinase (MMP)-9 signal. Interestingly, a depletion of CD44 leads to metabolic pathway changes resulting in redox status modification and Trolox (anti-oxidant) led to the recovery of the cancer cell functions. Based on our findings, the regulation of CCA progression and metastasis via the redox status-related Akt signaling pathway depends on the alteration of metabolic profiling synchronized by CD44.

## Introduction

Cholangiocarcinoma Cluster of differentiation 44 (CD44) is a family of transmembrane glycoproteins containing extracellular transmembrane and intracellular cytoplasmic domains [1]. Members of this family are alternatively spliced into several variant exon products of the extracellular domains as variant isoforms [2]. CD44 can be used as a cell surface marker in order to identify cancer stem-like cells (CSCs) in many cancer types [3–6]. The functional relevance of CD44 expression is associated with features of CSCs that are shared with normal stem or progenitor cells, such as the interaction with the corresponding niche [7, 8], the potential for cell migration and homing [9–11], the capacity for defense against ROS [12–14] and resistance to apoptosis [15–17]. A previous report suggests that the phosphorylation of CD44 can trigger intracellular signaling, particularly Akt protein, for supporting cell growth, motility and invasion [18]. Additionally, CD44 has been reported to promote epithelial-mesenchymal transition (EMT), which is an important process enhancing migration and invasion via Akt signaling [19]. The expression of the CD44 variant 8–10 (CD44v8-10) on the cell surface stabilizes xCT (a cystine-glutamate transporter) and contributes to glutathione (GSH) synthesis for ROS defense, leading to redox regulation in several cancers [12, 14, 20]. Although the functions of CD44 in a broad variety of cellular processes are mediated by several mechanisms, the redox status regulation of CD44 via ROS interacts directly with critical signaling molecules to initiate signal transduction. These include the phosphoinositide-3-kinase- (PI3K-) Akt pathway, which is a vital mechanism contributing to cancer development and metastasis [21, 22]. Taken together, the CD44 mediated Akt pathway not only plays an important role in cell proliferation, but is also crucial in cell migration and invasion, possibly via several mechanisms including redox regulation and phosphorylation.

Cholangiocarcinoma (CCA) is a cancer of the bile ducts that is generally characterized by its late diagnosis and fatal outcome [23]. Moreover, a significant positive association between the incidence of CCA from the cancer registry and liver fluke, *Opisthorchis viverrini* (Ov), infection is found in the northeast of Thailand [24]. Ov-induced chronic inflammation links to the onset of cholangiocarcinogenesis via pro-inflammatory cytokines (i.e. IL-6) and transcription factor NF-κB that control the oxidative stress response enzymes COX-2 and iNOS generating reactive oxygen (ROS) and nitrogen species (RNS). Moreover, an overproduction of ROS and RNS may lead to an alteration in redox status regulation via CD44v8-10 upregulation, which is a cancer stem-like cells (CSCs) marker during cholangiocarcinogenesis. This is supported by a previous report indicating that the redox status regulation of CCA cells depends on the expression of CD44v8-10 that is associated with the xCT function and is a link to the poor prognosis of patients [14]. Hence, CD44 positive cells might contribute to the damaged cells that enter the carcinogenesis process and enable cancer cells to resist anti-cancer treatment via regulating redox status. Most importantly, the role of CD44 and its molecular mechanism related to CCA progression, including proliferation, migration and invasion, is still unclear.

Therefore, this study aims to investigate function of CD44 on the cellular processes of CCA cells, including proliferation, migration, and invasion, as well as molecular mechanisms and metabolic profiling changes related to the ROS signaling pathway.

## Materials and methods

### Cell culture and reagents

The protocols of the present study were approved by the Ethics Committee for Human Research, Khon Kaen University (HE571283 and HE591002, respectively). The human cholangiocarcinoma cell lines KKU-213 and KKU-156 were purchased from the JCRB cell bank. Both cell lines were grown in DMEM medium (Gibco Life Technology, CA, USA), supplemented with NaHCO3, 100 units/ml penicillin, 100 ug/ml streptomycin and 10% fetal bovine serum at 37°C containing 5% CO2 in a humidified incubator. 6-hydroxy-2,5,7,8-tetramethylchromane-2-carboxylic acid (Trolox) is a water-soluble analog of vitamin E that protects against cell damage by oxidants (Sigma-Aldrich).

### Gene knockdown and establishing stable cell lines

The CD44 genes in KKU-213 and KKU-156 cells were silenced using lentivirus (Sigma-Aldrich) containing shRNAs for human CD44. Briefly, 1.6 x 10^4^ cells of KKU-213 and KKU-156 were seeded into a 48-well plate. A lentiviral construct was then transduced into the cell lines with DMEM containing hexadimethrine bromide (Sigma-Aldrich). A lentivirus containing shRNAs for a non-targeting gene was used as a control (Sigma-Aldrich). After 24 h transduction, KKU-213 and KKU-156 cells were treated with 1 μg/ml of puromycin (Sigma-Aldrich) for two weeks to establish stable clones of CD44 KD, and the expression of CD44 was confirmed using flow cytometry.

### Flow cytometry analysis

For flow cytometry (FACS) analysis, single-cell suspensions of control and CD44 KD cells were incubated with antibodies to CD44 (Invitrogen, CA, USA) for 15 min at 4°C. Apoptotic cells were excluded during flow cytometry by elimination of cells staining positive with propidium iodide (Sigma-Aldrich). Flow cytometry analysis was conducted with FACSCanto II (BD Bioscience, Khon Kean, Thailand) and analyzed with BD FACSDiva^™^ software.

### Western blot analysis

CCA cell lines with either transduced controls or with CD44shRNA (5x10^5^ cells per well) were seeded into cell culture dishes for 24 h and then the pellets of these cells were collected. The method was performed as previously described [25]. In brief, equal amounts of protein (15 μg) with SDS sample buffer were separated on 10% polyacrylamide gels by electrophoresis and transferred onto polyvinylidene difluoride membranes. The membranes were incubated with primary antibodies against total-Akt, phospho-Akt, total-mTOR, phospho-mTOR, p21, Bax, Bcl-2, E-cadherin, vimentin, and β-actin, overnight at 4°C. They were then incubated with secondary antibody for 1 h. Anti-total-Akt (1:1000, cat.no. AB59380), phospho-Akt (1:1500, cat.no. 9271S), total-mTOR (1:1000, cat.no.2983s), phospho-mTOR (1:1000, cat.no.2971s), Anti-cyclin D1 (1:1000, cat.no. 2922s) antibodies were purchased from Cell Signaling Technology, MA, USA. Anti-Bax (1:1000, cat.no.610983) and anti-E-cadherin (1:2000, cat.no.610182) were purchased from BD biosciences, NJ, USA. Anti-p21 (1:200, cat.no.sc397) was purchased from Santa Cruz Biotechnology, CA, USA and Bcl-2 (1:500, cat.no.12789-1-AP) was purchased from Proteintech, Manchester, UK. Anti-CDK4 (1:1000, cat.no. AB108357) and Anti-vimentin (1:1000, cat.no. AB92547) from Abcam, Cambridge, UK. Anti-β-actin (1:10000, cat.no. A5441) was purchased from Sigma Aldrich. The signals were analyzed using chemo luminescence enhancing with an ECL Prime Western Blotting Detection System (GE Healthcare, Little Chalfont, UK). β-actin expression was used as an internal control.

### Immunofluorescence analysis

CCA cell lines with either transduced controls or CD44shRNA (1x10^4^ cells/ml) were seeded into a slide chamber for 48 h and then fixed with 4% paraformaldehyde. The samples were exposed to 3% BSA and incubated with primary antibodies against p21(1:50), MMP-9 (1:200, cat.no. AF911, R&D system, Minneapolis, MN, USA), E-cadherin (1:200), and vimentin (1:200) overnight at 4°C. After washing with PBS, the samples were next incubated with Alexa Fluor 488 or 555-conjugated secondary antibodies (Invitrogen) and mounted in Hoechst 33342 (Invitrogen). The fluorescence signals were detected under a Zeiss LSM800 confocal laser scanning microscope (Carl-Zeiss, Oberkochen, Germany).

### Cell cytotoxicity analysis

The number of viable cells was evaluated with a Cell Titer-Glo luminescence cell viability kit (Promega, Madison, WI). In short, CCA cell lines with either transduced control or CD44shRNA (2x10^3^ cells per well) were plated into 96-black well plates for 24, 48, 72 and 96 h. The luminescence signal was detected on a SpectraMaxL microplate reader. instructions. Experiments were performed three times with three replicates per experiment.

### Migration assay

CCA cell lines with either transduced control or CD44shRNA (2x10^4^ cells per well) with serum free DMEM were placed on the upper layer of a cell permeable membrane and a solution containing 10% fetal bovine serum was placed below the cell permeable membrane for 24 h for KKU-213 and 48 h for KKU-156. Next, migrated cells were fixed with methanol and then stained with hematoxylin (Sigma-Aldrich). The number of cells that migrated through the membrane were counted as cells per field under a Microscope Axio Imager A2 (Carl-Zeiss, Oberkochen, Germany, located in Khon Kean, Thailand).

### Invasion assay

Corning^®^ BioCoat^™^ Matrigel^®^ Invasion Chamber, 8.0μm PET Membrane 24 well permeable supports packaged ready to use in Falcon^®^ Companion Plates were purchased from corning (cat.no.354480, Corning, NY, USA). Membranes from the invasion chamber were activated with serum free DMEM. CCA cell lines with either transduced control or CD44shRNA (2x10^4^ cells per well) with serum free DMEM were placed on the upper layer of a cell permeable membrane and a solution containing 10% of fetal bovine serum was placed below the cell permeable membrane for 24 h for KKU-213 and 48 h for KKU-156. Next, invasive cells were fixed with methanol and then stained with hematoxylin (Sigma-Aldrich, MO, USA). The number of cells that invaded through the membrane and matrix gel were counted as cells per field under a Microscope Axio Imager A2 (Carl-Zeiss, Oberkochen, Germany, located in Khon Kean, Thailand).

### The Trolox treatment assay

The KKU-213 CCA cell line with either transduced control or CD44shRNA (2x10^3^ cells per well) was treated with Trolox for 24 h. The number of viable cells was evaluated with a Cell Titer-Glo luminescence cell viability kit (Promega, Madison, WI). The luminescence signal was detected on a SpectraMaxL microplate reader.

In addition, the CCA KKU-213 cell line with either transduced control or CD44shRNA (2x10^4^ cells per well) with serum free DMEM with the absence or present of Trolox was placed on the upper layer of a cell permeable membrane and a solution containing 10% fetal bovine serum was placed below the cell permeable membrane for 24 h. Next, migrated cells were fixed with methanol and then stained with hematoxylin (Sigma-Aldrich). The number of migrated cells were counted as cells per field under a Microscope Axio Imager A2 (Carl-Zeiss, Oberkochen, Germany, located in Khon Kaen, Thailand).

### The collection of cellular metabolites

A total of 15x10^6^ cells of KKU-213 were counted, collected and washed with 1xPBS pH 7.4. Before homogenization, the cells was dipped into liquid nitrogen and then slowly thawed on ice. The cellular metabolites were extracted with methanol and chloroform; the extract was separated into 2 phases, a polar phase and a lipophilic phase after centrifugation at 1000 g for 15 min. The solvents were removed from the samples using a speed vacuum concentrator and the extracts were kept at –80 °C. Prior to NMR data acquisition, the polar cell extract was resuspended in 580 μl of D_2_O containing 0.1mM 3-trimethysilypropionic acid (TSP) (Cambridge Isotype Laboratories, Massachusetts, USA) as a chemical shift reference (δ = 0 p.p.m.). A total of 550 μl of the supernatant was transferred into an NMR tube with mm diameter after vortexing and centrifuging at 12000 g for 5 min.

### The collection of extracellular metabolites

The conditioned media of KKU-213 cells was collected after the cells were cultured for 48 h at 37°C under 5% CO_2_ in a humidified incubator. The free water samples were concentrated using a speed vacuum concentrator and the extracted samples kept at -80 °C. Before NMR acquisition, polar cell extraction was resuspended in 580 μl of D_2_O containing 0.1mM TSP (Cambridge Isotype Laboratories) as a chemical shift reference (δ = 0 p.p.m.). A total of 550 μl of the supernatant was transferred into an NMR tube after vortexing and centrifuging at 12000 g for 5 min.

### ^1^H NMR data acquisition

^1^H NMR analysis was performed using Bruker AVANCE III 400 MHz spectrometer at 300 K. All samples were analysed using standard 1-dimension pulse sequence (recycle delay-90°-t1-90°-tm-°-acquisition) with t1 and to 3 ms, tm to 10 ms, abd 90° pulse to 10 μs in 64 scans.

### Spectral data processing

The phase and baseline of all NMR spectra were adjusted in MATLAB software, and the TSP peak was set to 0 ppm. Data points within 0.6–9.0 ppm were kept and the water peak interval (4.5–5.0 ppm) was removed. The data were calculated according to a control spectrum after probabilistic quotient normalization (PQN).

### Metabolite identification and network analysis

Pseudo two-dimensional spectra were drawn based on preprocessed data using statistical total correlation spectroscopy (STOCSY) which indicates correlation between factors among each chemical shift [31, 32]. The ratio of false positives and the threshold of the correlation factor r were calculated. P-value was adjusted with Bonferroni false discovery rate (FDR) correction. The area under peaks was also integrated and used as relative concentration. The relative concentrations of metabolites were analyzed using log2 fold-changes using GraphPad Prism 5 (GraphPad Software, Inc., CA, US). The metabolites with log2 fold-changes were analyzed using Spearman correlation using R programming. The names of the metabolites and their HMDB ID were matched and network analysis was performed using MetaboAnalyst (www.metaboanalyst.ca/) [26]. The MBROLE2 system, accessible at http://csbg.cnb.csic.es/mbrole2 [27], was used for pathway analysis including metabolite-metabolite interactions.

### Statistical analysis

SPSS software version 17.0 (IBM Corporation, Armonk, NY) was used for statistical analysis of clinical and baseline data. The differences among each group of samples were analyzed using a t-test. The data were expressed as a graph of mean ± S.D. using Graph Pad prism 5. All analyses were two-tailed and P-values < 0.05 were considered statistically significant.

## Results

### CD44 depletion effects on cell proliferation

**Fig 1:**
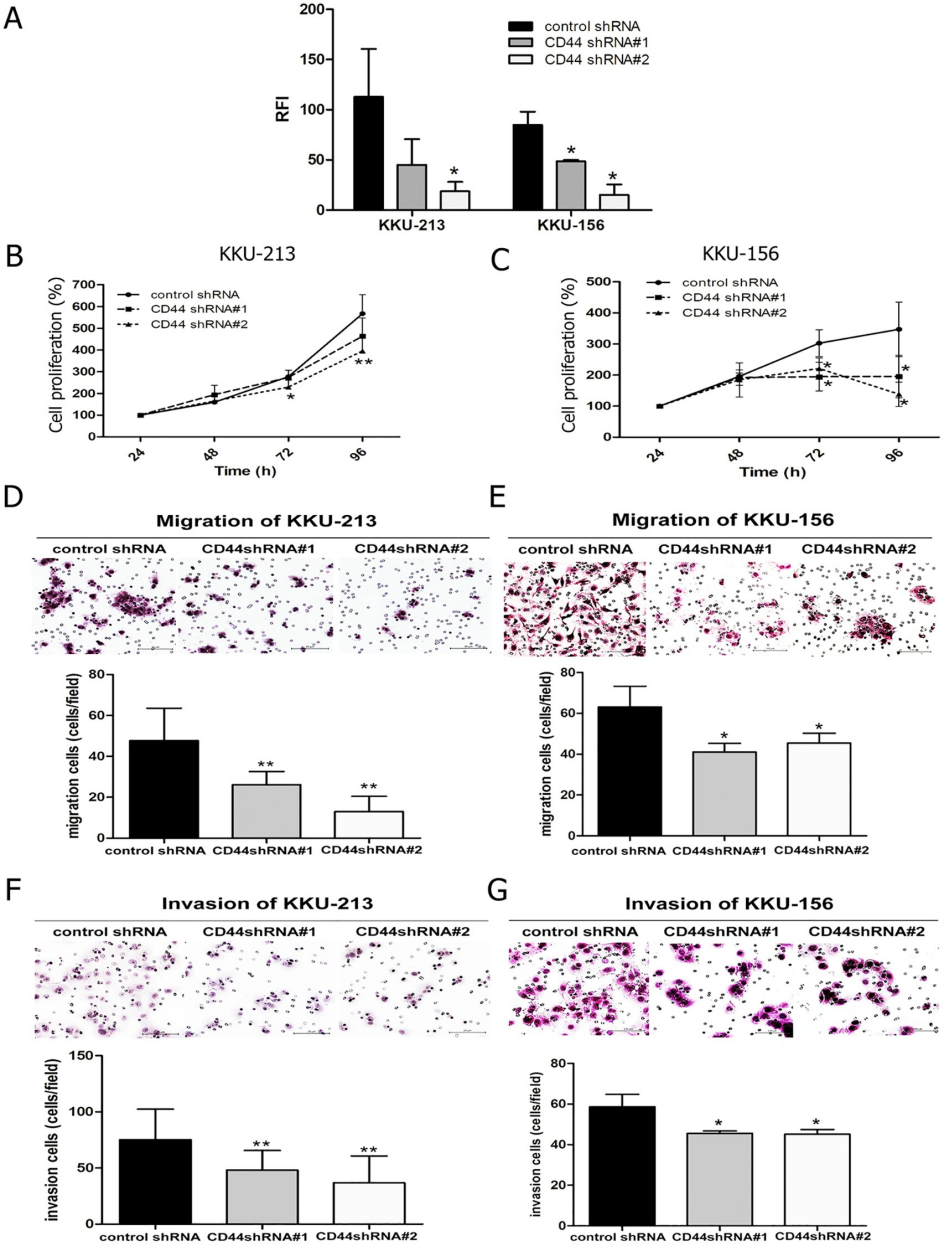
Knockdown of CD44 inhibited cell proliferation, migration and invasion. (A) Basal level of CD44 was highly expressed in both CCA cell lines and the signal of CD44 was reduced after lentiviral transduction of CD44shRNA. The fluorescent intensity of CD44 staining using flow cytometry represented as a relative fluorescence intensity ratio (RFI). (B) CCA proliferation was suppressed in a CD44 silencing of KKU-213 and (C) KKU-156 cells. (D) The migration of CCA cells with CD44 silencing was reduced in KKU-213 and (E) KKU-156 cell lines. (F) Suppression of CD44 expression shows that the number of invasive cells decreased in both KKU-213 and (G) KKU-156 cell lines. Data are expressed as mean ± S.D. from three-time independent experiments. *p < 0.05, **p < 0.01 from t- test analysis.

To study the function of CD44 on CCA cells, we carried out stable knockdown of CD44 and examined cell proliferation, migration and invasion. The evaluation of CD44 level using flow cytometry shows a high CD44 expression on cell surfaces in both CCA cell lines. Furthermore, stable knockdown in KKU-213 and KKU-156 CCA cell lines was performed using CD44shRNA to target two different sequences allowing the exploration of cellular functions including cell proliferation. Fig 1A shows that the level of CD44 was decreased in KKU-213 and KKU-156 after the knocking down by CD44shRNA in both targets. The levels of CD44 expression of KKU-213 were decreased to 45% and 18% of control in CD44shRNA target 1 (p = 0.0577) and 2 (p = 0.0263), respectively, and KKU-156 were reduced to 48% and 15%in both CD44shRNA target 1 (p = 0.0114) and target 2 (p = 0.0002), respectively.

In addition, the depletion of CD44 reduced proliferation at 72 and 96 h in both CCA cell lines (Fig 1B and 1C). The cell proliferationin percentage of CCA cells with transduction of CD44shRNA targeting sequence 2 was significantly reduced in KKU-213 cells at 72 h (p = 0.017) and 96 h (p = 0.035). Additionally, cell viability of CCA cells with transduction of CD44shRNA targeting both sequences 1 and 2 was significantly decreased in KKU-156 cells at 72h (p = 0.005 for sequence 1, p = 0.011 for sequence 2). The cell viability in KKU-156 CCA cells with transduction of CD44shRNA targeting sequence 2 was also significantly diminished at 96 h (p = 0.047).

### CD44 depletion effects on cell migration and cell invasion

CCA cell migration and invasion were also decreased in CD44-stable knockdown cells when compared with control shRNA-stable knockdown CCA cell lines in KKU-213 and KKU-156. Both cell types demonstrated that the migration of CCA cells with transduction of CD44shRNA targeting sequence 1 and 2 was significantly reduced in KKU-213 (p <0.001, p <0.001, respectively) and KKU-156 (p = 0.016, p = 0.046, respectively) (Fig 1D and 1E). Moreover, the invasion of CCA cells with transduction of CD44shRNA targeting sequence 1 and 2 was significantly decreased in KKU-123 (p = 0.006, p <0.001, respectively) and KKU-156 (p = 0.018, p = 0.007, respectively) (Fig 1F and 1G).

### Trolox recovers cell proliferation and migration after CD44 depletion via Akt activation

**Fig 2:**
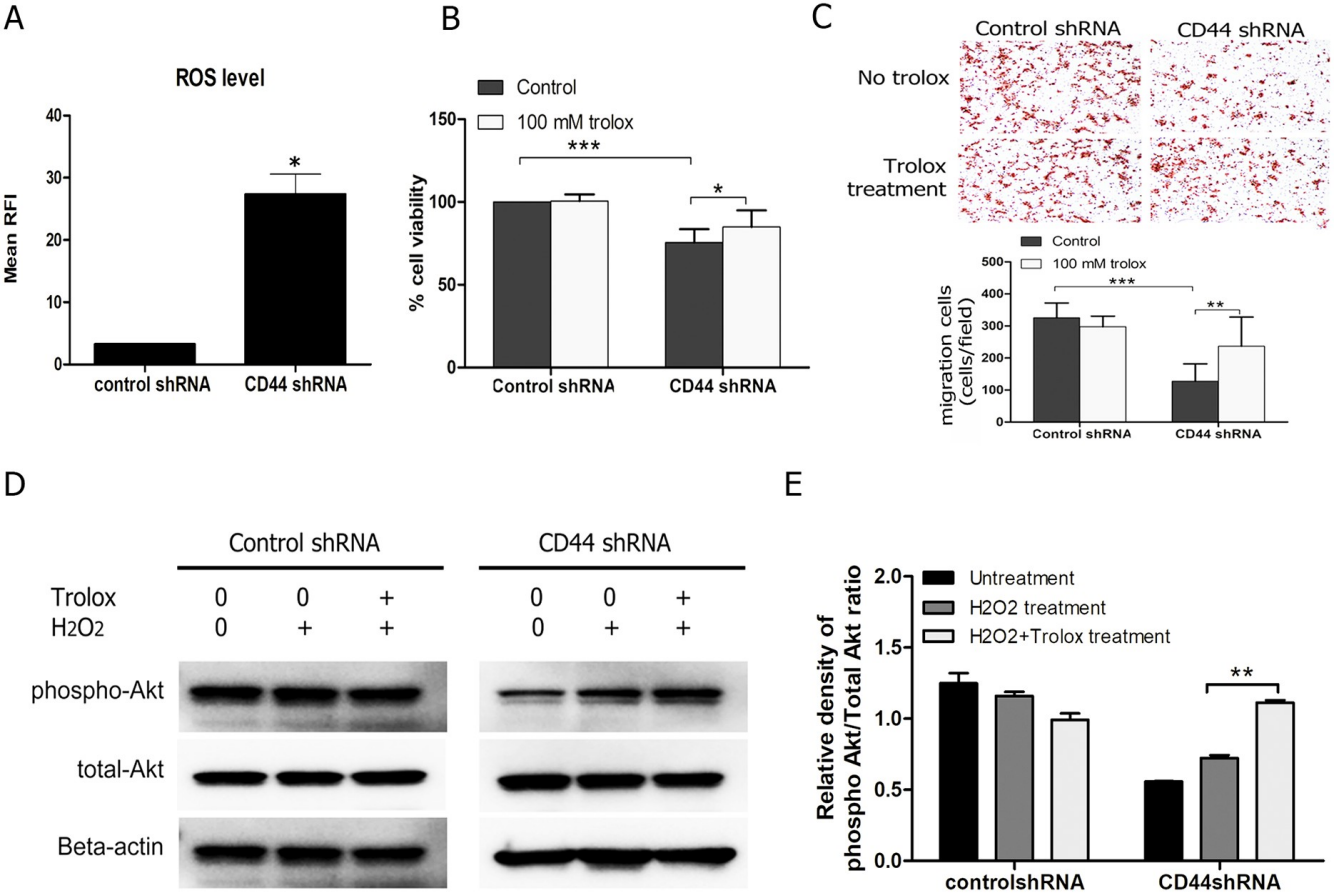
The presence of Trolox (ROS scavenger) led to the recovery of the the depletion of CD44. (A) Intracellular ROS level of CD44 silencing in CCA cells was increased. (B) CCA proliferation was suppressed in a CD44 silencing of KKU-213 and recover after Trolox treatment. (C) The migration of CCA cells with CD44 silencing was increased in the presence of Trolox. (D) Expression level of Akt was increased after Trolox treatment in CD44-silencing CCA cell lines, while control was not altered. (E) The optical density of protein expression using the western blot method shows that the phosphorylation of Akt. Data are expressed as mean ± S.D. from three-time independent experiments. *p < 0.05, **p < 0.01, ***p < 0.001 from t- test analysis.

The intracellular ROS level was increased in CD44 knockdown CCA cell (Fig 2A). We, therefore, tested the effect of Trolox (ROS scavenger) on cell viability and migration to confirm the reverse effect on these factors in silencing of CD44. The result demonstrates that Trolox could recover cell viability (Fig 2B) and migration in CD44 knockdown cells (Fig 2C). Interestingly, the phosphorylation of Akt was increased after Trolox treatment in CD44shRNA silencing cells, while, control cells were not changed (Fig 2D and 2E). The phospho-Akt protein was not altered after treatment with 100 μM hydrogen peroxide in the control, whereas CD44 knockdown cells was slightly upregulated. These findings indicate that CD44-mediated Akt activation and its downstream targets is associated with intracellular ROS change.

### The mechanisms of CCA progression is regulated by CD44 protein through the Akt signaling pathway

**Fig 3:**
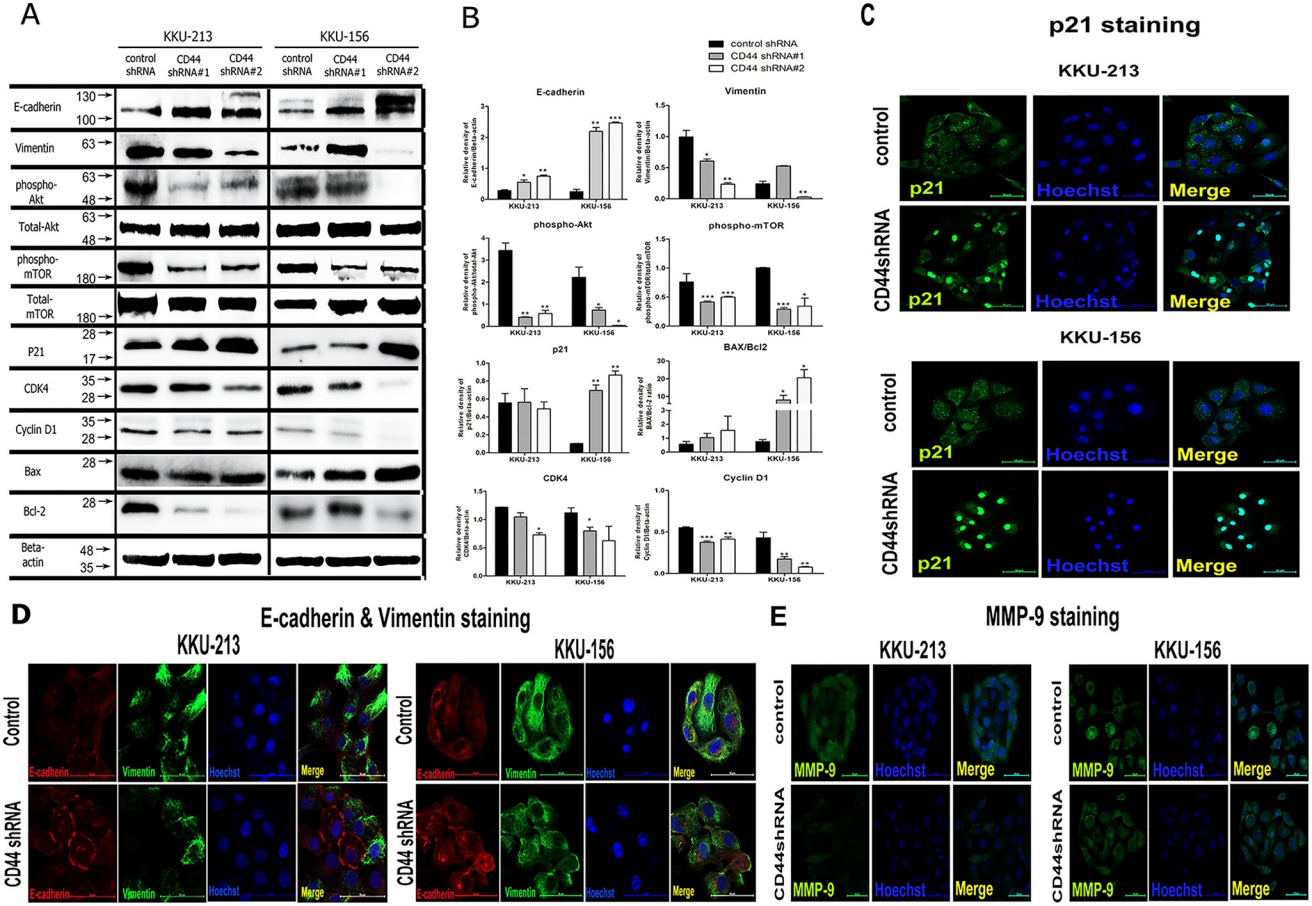
The molecular mechanism of CD44 regulated cancer progression including cell proliferation, migration and invasion, possibly through the Akt-mediated epithelial mesenchymal transition (EMT) pathway. (A) Western blot indicates that the downstream proteins of Akt that are involved in cell proliferation, migration and invasion were altered after CD44 silencing. (B) The optical density of protein expression using the western blot method shows that the phosphorylation of Akt and mTOR in CCA cells with CD44 depletion was lower than control cells, subsequent to an increase of the p21 and Bax/Bcl-2 ratio, as well as alteration of epithelial mesenchymal transition (EMT) markers (E-cadherin was increased while vimentin was decreased), resulting in an inhibition of cell proliferation, migration and invasion. (C) The translocation of p21 (cell cycle inhibitor) into the nuclear part of CCA cells with CD44 depletion was increased in KKU-213 and KKU-156, causing the inhibition of cancer cell proliferation. (D) Co-localization of E-cadherin and vimentin using immunofluorescence staining shows an increase in membranous E-cadherin and a decrease in cytoplasmic vimentin with suppression of cancer cell migratory ability in KKU-213 and KKU-156. (E) A reduced expression of MMP-9 was demonstrated in the CD44 depleted-CCA cells, contributing to a suppression of cancer cell invasion ability. Data are represented as mean ± S.D from two-time independent experiments. *p < 0.05, **p < 0.01 from t- test analysis.

Our findings demonstrated that CD44 protein appeared to play important roles in cancer progression. We next examined the molecular mechanisms of CD44 contributing to cell proliferation (Akt, mTOR, p21, CDK4, cyclin D1, Bax, Bcl-2), migration (E-cadherin, vimentin) and invasion (MMP-9) using the western blotting analysis and the localization of protein expression was also conducted using immunofluorescence staining. Our results revealed the downregulation of phosphorylation of Akt and its downstream target (mTOR) in KKU-213 and KKU-156 cells, with CD44 depletion in both targets (Fig 3A and 3B). Consequently, p21 cell cycle inhibitor was upregulated and translocated to the nucleus after CD44 depletion (Fig 3C), potentially leading to cell cycle arrest possibly *via* CDK4 and cyclin D1 depletion (Fig 3A). These circumstances might trigger apoptosis pathway through an increase of pro-apoptotic protein (Bax) and a decrease in pro-survival protein (Bcl-2) (Fig 3A and 3B). Moreover, the investigation of epithelial mesenchymal transition (EMT) markers using immunoblotting showed that E-cadherin was increased while vimentin was decreased after CD44 depletion of KKU-213 and KKU-156 cells (Fig 3A and 3B). In addition, the co-localization of E-cadherin and vimentin revealed that the upregulation of E-cadherin and the downregulation of vimentin are found in CD44 silencing of KKU-213 and KKU-156 cells (Fig 3D). Furthermore, the immunofluorescence of MMP-9 shows that silencing of CD44 in both CCA cell lines was slightly decreased (Fig 3E). Therefore, the progression of CCA cells, including proliferation, migration and invasion, was potentially driven by CD44-mediated Akt activation and its downstream targets.

### The alteration of metabolic profiling in CD44-suppressed CCA cells

**Fig 4:**
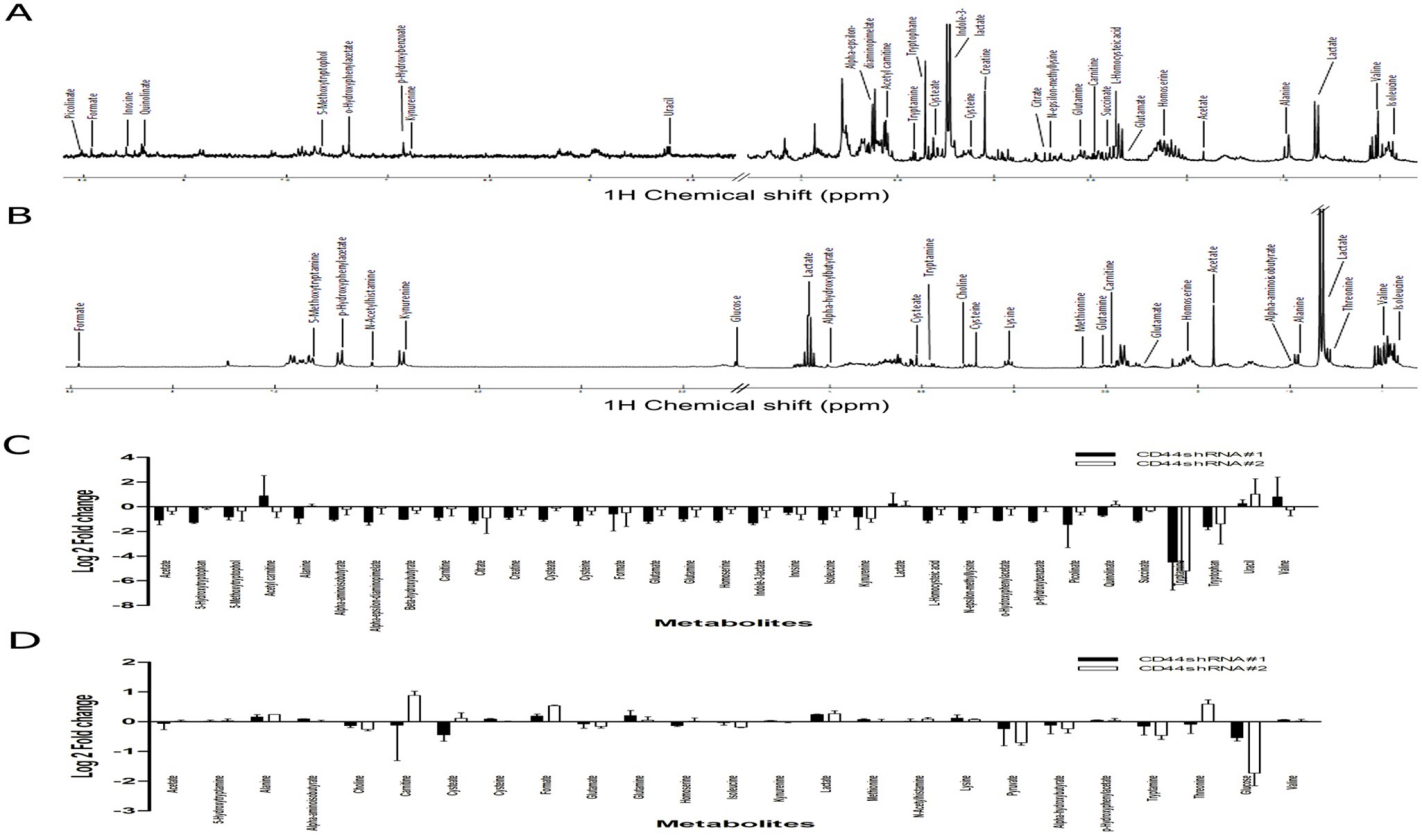
Representative spectra and peak assignment. (A) NMR spectra of CCA cells and (B) conditioned media are presented.

CD44 protein plays important roles in cancer progression and metastasis possibly *via* Akt activation and its downstream targets. These alterations might derive from metabolic changes in both intracellular and extracellular metabolites. Therefore, we next identified the representative metabolites of CCA cells and conditioned media after interrupting CD44, and determined the changes in metabolic profiles using the NMR spectroscopic analysis. The metabolite assignment was confirmed with ChenomxNMR Suite software, STOCSY and HMDB. Fig 4 shows the spectra of cellular metabolites and conditioned media, while chemical shifts and corresponding groups in cells and conditioned media are summarized in Tables 1 and 2.

**Table 1:** Intracellular metabolite profiling of CCA cells.

Metabolites	Chemical shift (ppm)	Control shRNA		CD44shRNA#1		p-value	CD44shRNA#2		p-value
		Mean	SD	Mean	SD		Mean	SD	
**Acetate**	1.91(s); 1.92(s)	667128	29182	322165	83143	0.01	523787	91557	0.10
**5-Hydroxytryptophan**	3.23(dd); 3.41 (dd); 4.02(dd); 6.87(d); 6.88(d); 7.14(s); 7.28(s); 7.41(d)	194033	61584	80409	4554	0.08	182747	15643	0.78
**5-Methoxytryptophol**	0(d); 3(t); 3.88 (t); 3.9(s); 7.23(s); 7.26(s); 7.44(d)	117197	57916	66958	11452	0.27	102121	52028	0.75
**Acetyl carnitine**	2.14(s); 2.51(dd); 2.65(dd); 3.19(s); 3.61(d); 3.85(dd)	4932913	1500772	12747610	10214031	0.32	3855501	1225065	0.39
**Alanine**	1.48(d); 3.79(q)	884189	11149	479563	141872	0.04	922756	107598	0.60
**Alpha-aminoisobutyrate**	1.5(s)	1477933	482048	722090	54877	0.11	1318779	404607	0.68
**Alpha-epsilon-diaminopimelate**	1.49(m); 1.92(m); 3.76(m)	2559788	102200	1099715	202376	0.00	2451182	781234	0.83
**Beta-hydroxybutyrate**	1.2(d); 2.31(dd); 2.41(dd); 4.16(d)	971240	141569	480493	6691	0.03	798586	133032	0.20
**Carnitine**	2.44(dd); 3.23(s); 3.43(m)	849746	378844	467475	73459	0.22	804228	313848	0.88
**Citrate**	2.54(d); 2.66(d)	659169	56028	303781	48107	0.00	437905	303282	0.33
**Creatine**	3.04(s); 3.93(s)	4488821	693115	2462603	256711	0.03	3867877	1136739	0.47
**Cysteate**	3.29(dd); 3.55(dd);4.1(dd)	2220896	317704	1082841	99616	0.02	2068610	313045	0.59
**Cysteine**	3.03(dd);3.1(dd);3.97(t)	1657370	121969	760299	189833	0.00	1328734	282695	0.17
**Formate**	8.46(s)	149621	37042	126768	92004	0.72	127216	81669	0.70
**Glutamate**	2.1(m); 2.36(d);3.77(t)	2892800	462506	1282207	152812	0.02	2494610	764212	0.49
**Glutamine**	2.14(m); 2.46(m); 3.77(t)	754192	346014	380884	46926	0.20	662298	244858	0.73
**Homoserine**	2.03(m); 2.16(m); 3.79(m)	1096835	130230	510577	51023	0.01	956709	219972	0.41
**Indole-3-lactate**	3.08(dd); 3.26(d); 4.34(m);7.17(t); 7.27(t); 7.27(s);7.51(d);7.76(d)	1710004	482532	690074	71220	0.06	1455508	559470	0.58
**Inosine**	3.85(dd); 3.92(dd); 4.28(q); 4.44(t); 6.1(d); 8.24(s); 8.34(s)	246740	51512	177777	19628	0.13	164575	47692	0.11
**Isoleucine**	0.94(t); 1.01(d); 1.26(m); 1.48(m); 1.98(m); 3.68(d)	898136	309798	435585	101024	0.11	743661	245233	0.54
**Kynurenine**	3.72(d); 4.16(t); 6.82(t); 6.89(d);7.43(t); 7.89(d)	127932	32143	84013	50392	0.28	67310	13988	0.07
**Lactate**	1.33(d); 4.11(q)	3895019	217778	5170794	2740323	0.50	4218674	1084372	0.66
**L-Homocysteic acid**	2.32(m); 3.06(m); 3.96(t)	967600	157497	453590	62624	0.02	855886	252149	0.56
**N-epsilon-methyllysine**	2.71(s); 3.06(t); 3.76(t)	725033	97490	344629	54387	0.01	706893	198062	0.90
**o-Hydroxyphenylacetate**	3.54(s); 6.93(d); 6.95(t); 7.19(dd); 7.22(td)	618444	241470	284563	6159	0.14	565173	189185	0.78
**p-Hydroxybenzoate**	6.92(d); 7.81(d)	450376	214335	201034	13567	0.18	451751	113195	0.99
**Picolinate**	7.54(t); 7.92(d); 7.96(t); 8.57(s)	229509	139965	127654	107874	0.38	171037	27389	0.55
**Quinolinate**	7.45(q); 7.9(d); 8.02(d)	264691	119222	163173	9661	0.28	302347	60839	0.66
**Succinate**	2.41(s)	983427	70790	450228	37046	0.00	786386	36911	0.02
**Tryptamine**	3.18(t); 3.35(t); 7.21(t); 7.29(t); 7.34(s); 7.56(d); 7.71(d)	84715242	78963921	6567768	5928184	0.23	2655573	1589092	0.21
**Tryptophan**	3.31(dd); 3.49(dd); 4.06(dd); 7.21(t); 7.29(t); 7.33(s); 7.55(d); 7.74(d)	3012009	390483	973462	156376	0.01	1601840	1278019	0.19
**Uracil**	5.81(d); 7.54(d)	31847	12677	38780	8189	0.48	79705	54489	0.26
**Valine**	0.99(d); 1.04(d); 2.28(dd); 3.62(d)	1375559	469238	3327517	2632835	0.33	1188867	389537	0.62

*(S) singlet; (d); doublet; (t) triplet; (q) quartet; (dd) doublet of doublet; (m) multiplet.

Data are expressed as mean ± S.D. from two-time independent experiments. p < 0.05 from t- test analysis.

**Table 2:** Extracellular metabolite profiling of CCA cells.

Metabolites	Chemical shift (ppm)	Control shRNA		CD44shRNA#1		p-value	CD44shRNA#2		p-value
		Mean	SD	Mean	SD		Mean	SD	
Acetate	1.91(s); 1.92(s)	80621411	29581977	77793510	11296723	0.89	81684694	1908510	0.96
5-Hydroxytryptamine	3.12(t); 3.31(t); 7.1(s); 7.3(s); 7.42(d)	9453840	309635	9565075	230053	0.65	9643188	389248	0.55
Alanine	1.48(d); 3.79(q)	24838420	3043193	27763521	1430727	0.23	29358909	31434	0.12
Alpha-aminoisobutyrate	1.5(s)	7551400	2223	8064715	38533	0.00	7611648	190329	0.64
Choline	3.21(s); 3.52(m); 4.07(m)	19054547	711747	17442822	831861	0.06	15927649	539928	0.00
Carnitine	2.44(dd); 3.23(s); 3.43(m)	7928277	6230001	8862367	5926882	0.86	14657051	1378963	0.20
Cysteate	3.29(dd); 3.55(dd);4.1(dd)	14760560	1111762	10911037	1569558	0.03	16015323	2084060	0.42
Cysteine	3.03(dd);3.1(dd);3.97(t)	5168590	114695	5496504	59810	0.02	5173434	80343	0.96
Formate	8.46(s)	992562	200567	1131645	50819	0.35	1436960	27825	0.06
Glutamate	2.1(m); 2.36(d);3.77(t)	10139242	1235737	9646621	1021934	0.62	9076097	391355	0.27
Glutamine	2.14(m); 2.46(m); 3.77(t)	5055280	941146	5841647	686711	0.31	5238564	406770	0.78
Homoserine	2.03(m); 2.16(m); 3.79(m)	28531052	378523	26039497	656844	0.01	28854845	2250261	0.83
Isoleucine	0.94(t); 1.01(d); 1.26(m); 1.48(m); 1.98(m); 3.68(d)	31577921	2818006	30878412	1732803	0.74	27638507	354893	0.13
Kynurenine	3.72(d); 4.16(t); 6.82(t); 6.89(d);7.43(t); 7.89(d)	10828166	124376	11046765	75891	0.07	10822015	254377	0.97
Lactate	1.33(d); 4.11(q)	483437293	45780472	571290808	2378439	0.08	582492477	39725542	0.05
Methionine	2.14(s);2.16(m);2.65(t);3.86(t)	2918861	12539	3071895	63100	0.05	2944615	139671	0.78
N-Acetylhistamine	2.84(t);3.45(m);7.03(s);7.95(s)	3893415	166750	3940131	212722	0.78	4123136	156388	0.16
Lysine	1.48(m); 1.73(q); 1.91(m); 3.03(t); 3.76(t)	15716527	1132939	17103076	1293122	0.24	16508687	228739	0.35
Pyruvate	2.38(s)	14941174	4389969	13333639	5040858	0.70	9174226	566034	0.15
Alpha-hydroxybutyrate	0.9(t); 1.7 (m); 4.0(dd)	11290706	2058758	10534176	2090266	0.68	9589419	995065	0.29
p-Hydroxypheny-lacetate	3.45(s);6.87(d);7.17(d)	12081082	148156	12500655	112314	0.02	12403107	562972	0.43
Threonine	1.33(d);3.59(d);4.26(dq)	5472008	585886	4988499	1017401	0.52	3970536	349497	0.03
Tryptamine	3.18(t); 3.35(t); 7.21(t); 7.29(t); 7.34(s); 7.56(d); 7.71(d)	55098590	19499839	52738921	11129957	0.87	83057465	8047978	0.12
Glucose	3.69(m);3.82(m);8.78(s)	5459404	2440785	3754747	276885	0.35	1695742	487485	0.11
Valine	0.99(d); 1.04(d); 2.28(dd); 3.62(d)	38237289	338812	39865495	239507	0.00	38790628	1651787	0.62

(s) singlet; (d); doublet; (t) triplet; (q) quartet; (dd) doublet of doublet; (m) multiplet.

Data are expressed as mean ± S.D. from two-time independent experiments. p < 0.05 from t- test analysis.

By integrating the area under the peaks corresponding to signals of intrerest, the relative concentration of metabolites between the control sample and CD44 knockdown samples was determined as log2 fold changes (S1 and S2 Tables). Our results revealed intra-cellular thirty-three metabolites, whereas conditioned media contained twenty-five metabolites. We found that relatrive concentrations of acetate, citrate, creatine, cysteate, cysteine, glutamate, glutamine, homoserine, isoleucine, l-homocysteic acid, succinate, tryptamine, tryptophan and uracil were significantly altered. Interestingly, decreased levels of intracellular cysteine, glutamine, and homoserine after CD44 depletion were inversely associated with the level of extracellular metabolites. Therefore, the alteration of metabolic profiles, especially amino acid related-glutathione synthesis, was driven by CD44 and may contribute to CCA progression.

### Correlation of metabolite profile in CD44-silencing CCA cells

**Fig 5:**
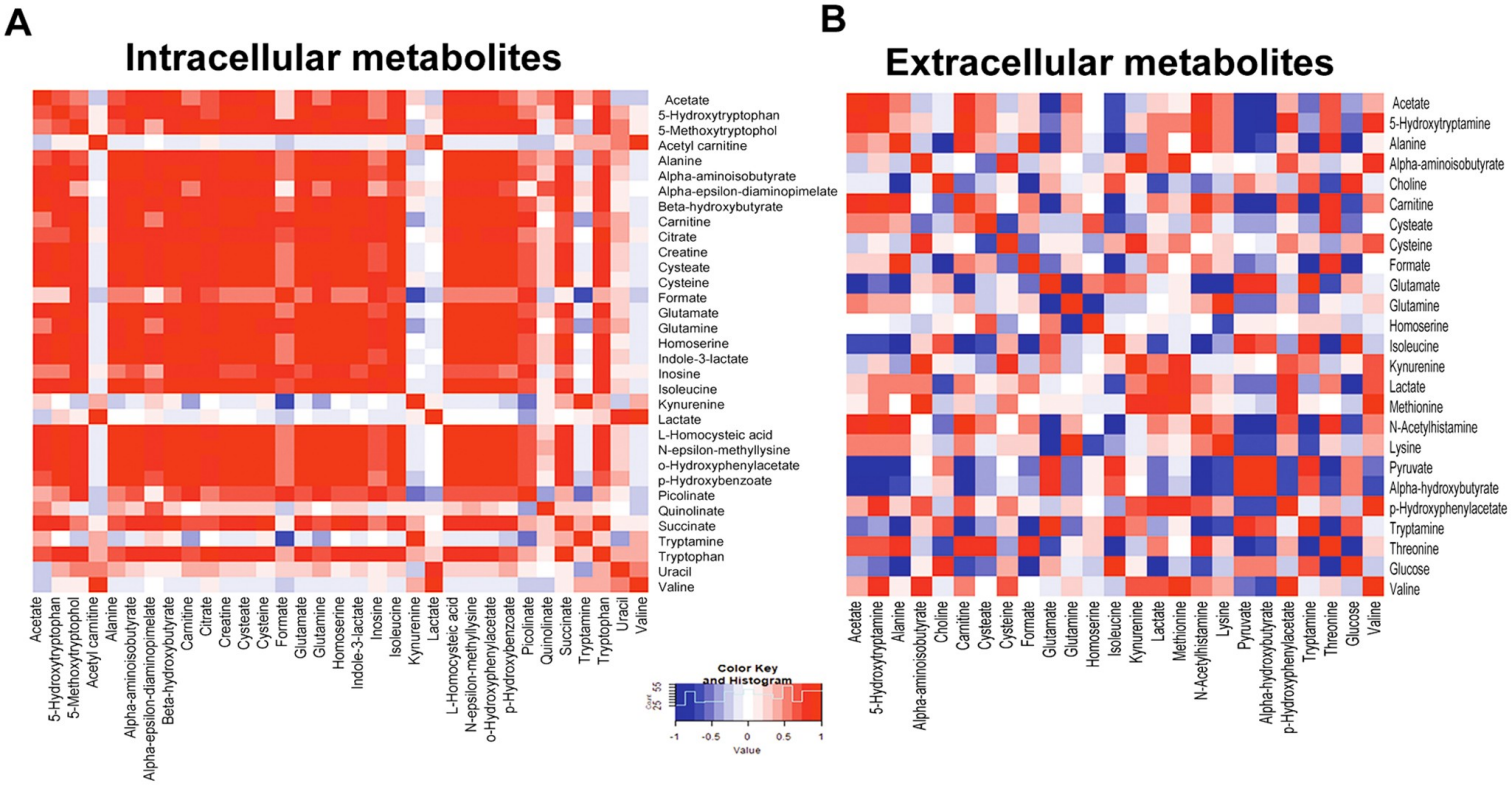
correlation between compounds shown as a heat-map. (A) a heat-map correlation of intracellular metabolites (B) a heat-map correlation of extracellular metabolites. Data are represented as mean ± S.D. from two-time independent experiments.

As the alteration in metabolites occurred after CD44 depletion, the association between each metabolite might be of importance. Hence, we next examined the metabolite inter-correlations using heatmap. The graphical output containing the metabolome view is presented in Fig 5A and 5B. Our figures show the heatmap correlation of each intracellular and extracellular metabolite from CCA cells. The association of metabolites after CD44 suppression was found in both the intra- and extra- cellular metabolic pathways, especially in glutathione synthesis and energy metabolism such as amino acid metabolism, and TCA cycle. For example, CD44-knockdown cells showed a decreased level of cysteine, a precursor of glutathione synthesis, directly associates with reduction of energy metabolism related-metabolites including acetate, carnitine, and succinate. In addition, this may trigger amino acid catabolism and decreased levels of amino acid and derivatives including creatine, alanine, glutamate, glutamine, homoserine, and tryptophan were clearly observed. Extracellularly, several compounds are inversely associated, for example, an increase in glutamine was related to the reduction of amino acid and its intermediate metabolites including glutamate, homoserine, pyruvate, alpha-hydroxybutyrate, and tryptamine. A high level of cysteine was correlated with reducing of cystease, formate and threonine. Moreover, a decrease in glutamate was associated with a low level of isoleucine.

### Suppression of the CD44 altered metabolic pathway in CCA cells

**Fig 6:**
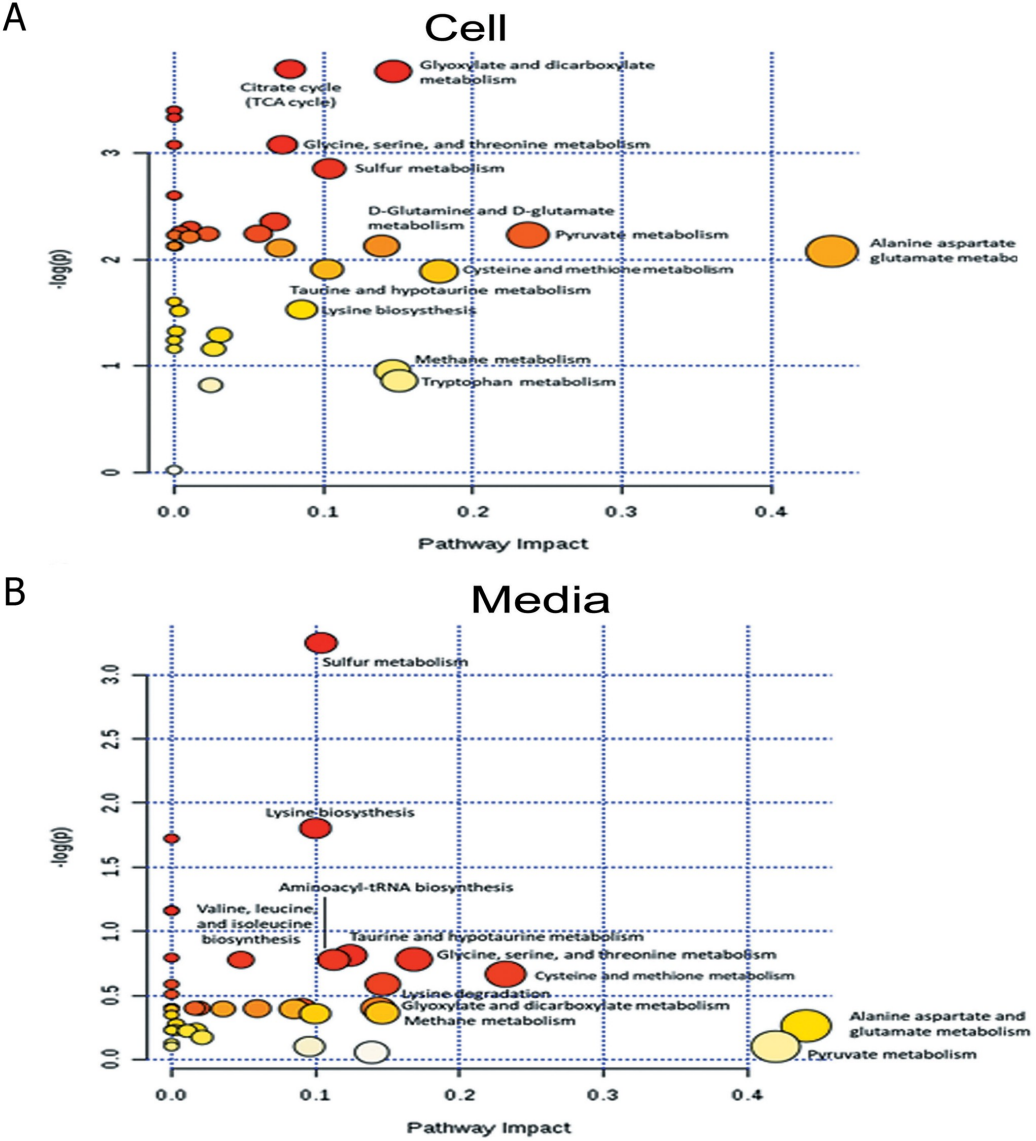
The influence pathway graph generated by MetScape. (A) the pathway impact of intracellular metabolites (B) the pathway impact of extracellular metabolites. Data are represented as mean± S.D. from two-time independent experiments.

**Fig 7:**
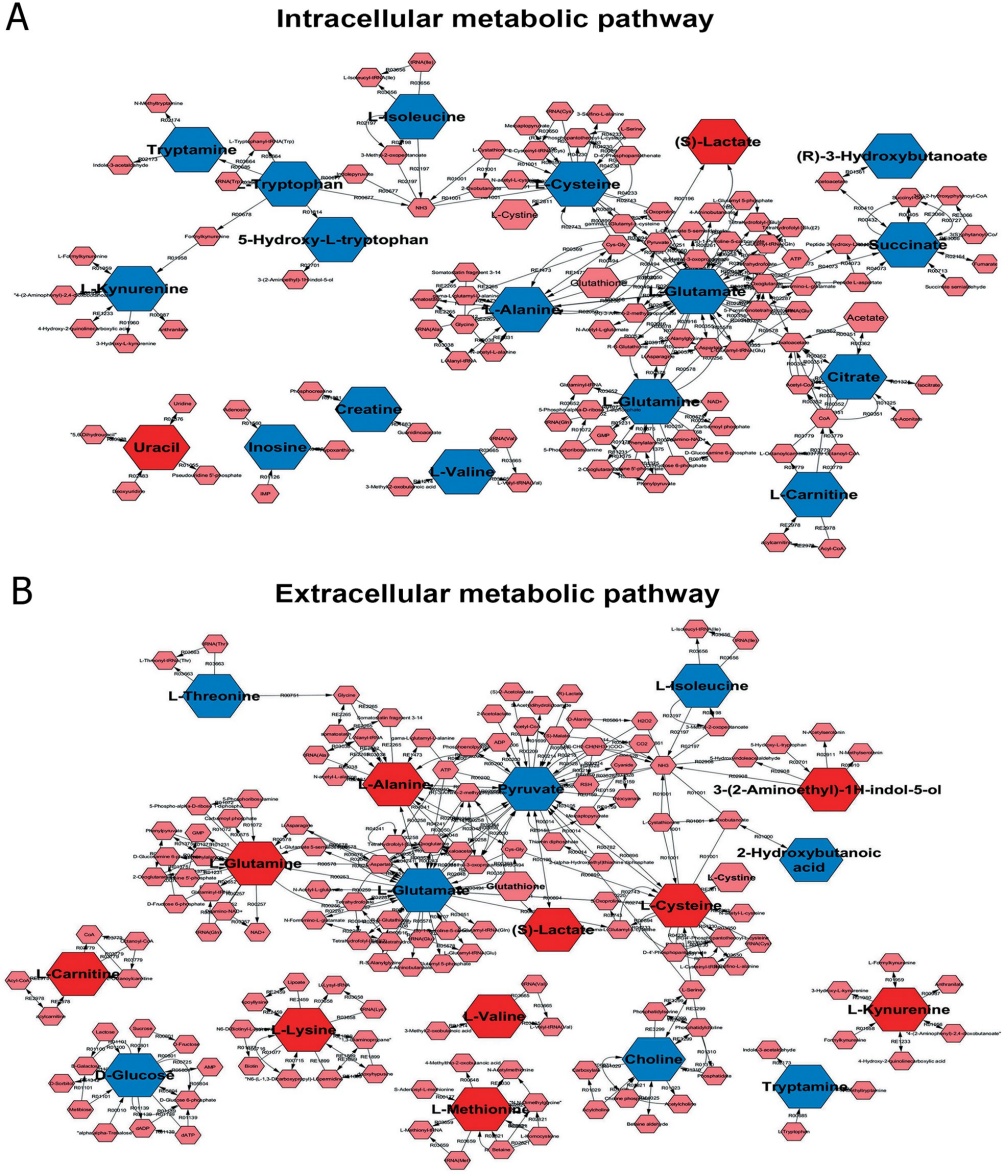
The network map generated by MetScape. (A) the related network map of intracellular metabolites (B) the related network map of extracellular metabolites. Red box indicates increased metabolites in CD44-knockdown cells and blue box indicates decreased metabolites. Data are represented as mean± S.D. from two-time independent experiments.

**Fig 8:**
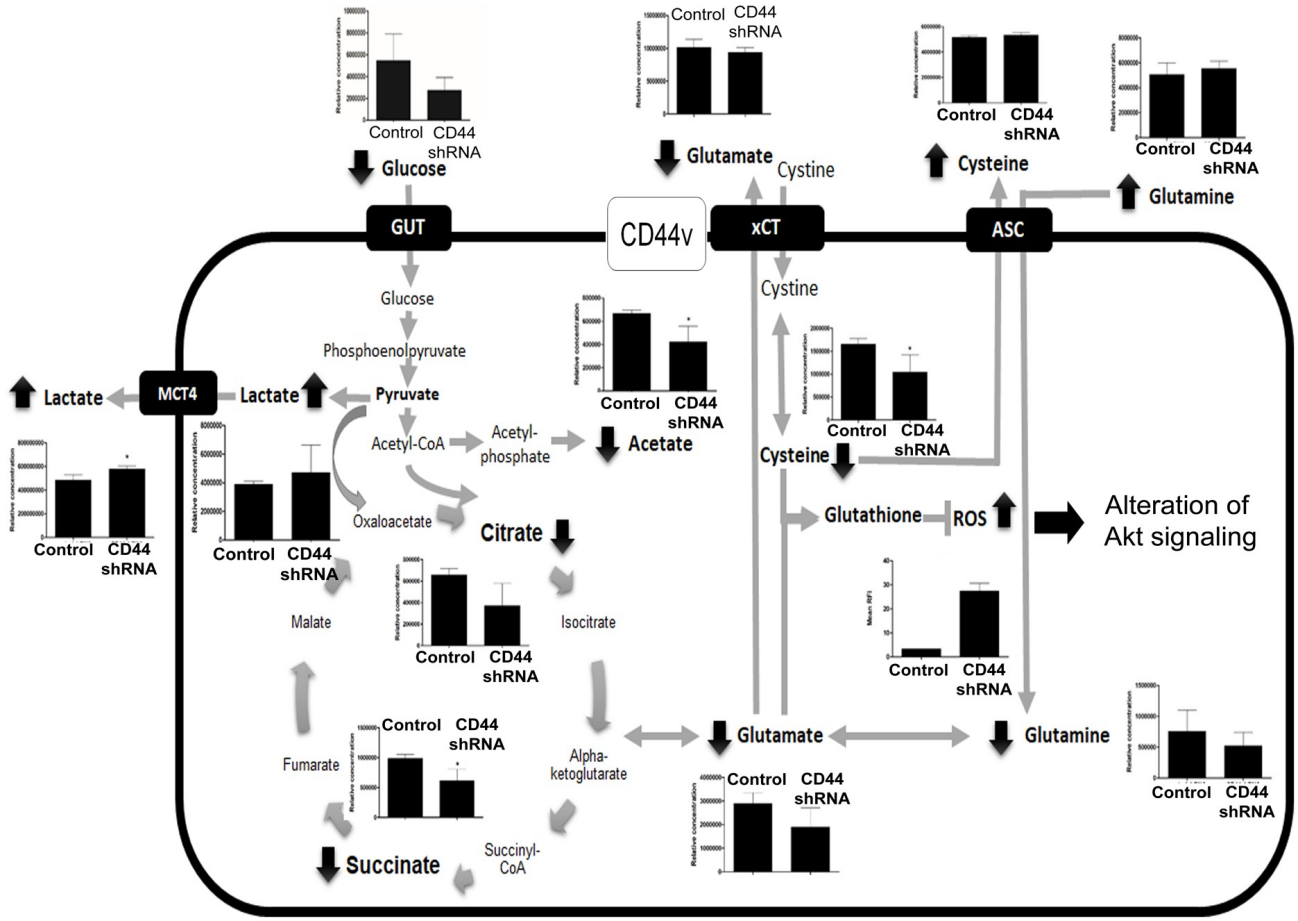
Proposed altered pathways in CD44-depleted CCA cell. Alteration of amino acid uptake after CD44 depletion leads to increasing ROS and Akt signaling alteration and metabolic changes. Data are represented as mean ± S.D. from two-time independent experiments. *p < 0.05 from t- test analysis.

Based on the relationship between the metabolites, network analysis and pathway enrichment analysis using MBRole showed significantly related pathways (Fig 5 and Table 3): these were alanine, aspartate and glutamate metabolism, pyruvate metabolism, cysteine and methionine metabolism, glyoxylate and dicarboxylate metabolism, citrate cycle (TCA cycle), sulfur metabolism, glycine, serine and threonine metabolism, D-glutamine and D-glutamate metabolism, and tryptophan metabolism (Fig 6A and 6B; Table 3). Fig 7A and 7B show the network pathway for intracellular compounds that were significantly associated with extracellular compounds, including cysteine, glutamine, and homoserine. Taken together, we conclude that the depletion determined by CD44 altered extra- and intra-cellular metabolic profiles of CCA cells, leading to increased levels of ROS, particularly due to the alteration of amino acids such as cysteine, glutamate, and glutamine, that is driven by CD44-mediated Akt activation and its downstream targets contributing to CCA progression (Fig 8).

**Table 3:** Influenced pathways generated from MBRole by enrichment analysis (adjusted p < 0.05).

ID Annotation (MAP ID)	Pathway	p-value	Adjusted p-value	In background	In set
1100	Metabolic pathways	1.93E-11	1.43E-09	1455	24
970	Aminoacyl-tRNA biosynthesis	9.34E-08	3.46E-06	75	7
430	Taurine and hypotaurine metabolism	3.22E-06	5.96E-05	20	4
250	Alanine, aspartate and glutamate metabolism	6.96E-06	1.03E-04	24	4
380	Tryptophan metabolism	6.30E-05	6.66E-04	81	5
2010	ABC transporters	1.05E-04	9.29E-04	90	5
920	Sulfur metabolism	1.13E-04	9.29E-04	18	3
260	Glycine, serine and threonine metabolism	1.26E-04	9.32E-04	49	4
270	Cysteine and methionine metabolism	2.14E-04	1.44E-03	56	4
910	Nitrogen metabolism	3.49E-04	2.15E-03	26	3
770	Pantothenate and CoA biosynthesis	3.91E-04	2.23E-03	27	3
620	Pyruvate metabolism	6.52E-04	3.22E-03	32	3
630	Glyoxylate and dicarboxylate metabolism	1.67E-03	6.39E-03	44	3
471	D-Glutamine and D-glutamate metabolism	1.84E-03	6.39E-03	12	2
20	Citrate cycle (TCA cycle)	5.17E-03	1.47E-02	20	2
290	Valine, leucine and isoleucine biosynthesis	1.00E-02	2.39E-02	28	2
10	Glycolysis / Gluconeogenesis	1.22E-02	2.66E-02	31	2
480	Glutathione metabolism	1.80E-02	3.33E-02	38	2
280	Valine, leucine and isoleucine degradation	2.08E-02	3.75E-02	41	2

The compounds from all metabolites in the pathway are listed; the **set** is the actually matched number from the user uploaded data; the p-value is the original p-value calculated from the enrichment analysis.

## Discussion

In the present study, we demonstrated that the molecular targets of CD44 are related to redox status regulation and inhibition of CCA cell proliferation, migration and invasion *via* activation of the Akt pathway. Although the functions of CD44 and its variants in cancer development have been well studied, the obvious molecular mechanism of CD44 related to redox status regulation contributing to CCA progression and metastasis is yet to be elucidated. Previously, it was shown that RNA interference-mediated depletion of CD44 and its variants, causing the reduction of xCT, leads to a decrease of intracellular glutathione and an increase in the level of intracellular ROS in CCA cells. Signaling pathways are frequently regulated by ROS through the interactions with key signaling molecules affecting a variety of cellular processes such as proliferation, metabolism, differentiation and survival, including PI3 kinase (PI3K)-protein kinase B (Akt) serine/threonine kinases [28]. ROS does not only activate PI3K directly to amplify its downstream signaling, but also concurrently inactivates the phosphatase and tensin homolog (PTEN), which inhibits the activation of Akt *via* oxidizing cysteine residues within the active center [29]. Furthermore, the phosphorylation of the CD44 intracellular domain can directly trigger intracellular signaling, particularly tyrosine kinase, including the Akt protein, for supporting growth, motility and invasion in many types of cancer [18], including breast [30, 31], prostate [32], and colon cancers [33].

The Akt pathway, or PI3K-Akt pathway, is a signal transduction pathway that promotes survival and growth in response to extracellular signals. Akt successively promotes the activation and transcription of its target genes (GSK3, FOXO, BAD, mTOR1, and p53), which are involved in growth, motility and invasion [34–37]. Thus, we hypothesize that the CD44 function is driven by Akt activation and its downstream effects, potentially *via* stabilizing xCT on membranes for regulating ROS signaling and phosphorylation of the CD44 intracellular domain.

Our data demonstrated that the RNA interference-mediated depletion of CD44 and its variants caused a decrease in cell proliferation, cell migration and cell invasion. Moreover, the inhibited CCA cell proliferation after silencing of CD44 can be recovered using the Trolox-vitamin E analog (data not shown). This indicates that the regulation of cell proliferation is associated with CD44 *via* the ROS-mediated cell signaling pathway. Moreover, the deletion of CD44 reduces the phosphorylation of Akt and mTOR, resulting in an increase of p21 (cell cycle inhibitor). Consequently, the pro-apoptotic proteins Bax was increased, while the anti-apoptotic protein Bcl-2 was reduced. Hence, we conclude that the inhibition of cell proliferation is possible through the ROS-Akt signaling pathway mediated p21 cell cycle inhibition, leading to an alteration in apoptosis related-proteins. Although the expression of Akt phosphorylation was increased in H_2_O_2_ treatment of CD44 silencing cells that might be involved with the imbalance of the redox status, the activation of Akt in CCA was regulated not only by the redox system, but also many other factors such as cytokines, growth factors, and bile acid [38]. Previously, it was demonstrated that the PI3K/AKT pathway in CCA is also stimulated by PTEN inactivation [39]. Currently, cell cycle progression is regulated by cyclin-dependent kinase (CDK) inhibitors such as p21Cip1, which are indirectly and directly controlled by Akt [40]. In addition, the cAMP response element binding protein (CREB; promoting cell survival) is phosphorylated by Akt at Ser133, stimulating recruitment of CREB-binding protein (CBP) to the promoter of target genes, especially Bcl-2 [41].

In addition to its role in cell growth, our results demonstrated that cell migration and invasion are decreased after CD44 silencing in CCA cells. Previous studies suggested that CCA patients with high expression of CD44 had a poor prognosis [14, 42], and an *in vitro* study of the depletion of CD44 showed that an inhibition of cell invasion and cell migration was seen in CD44 depleted CCA cells [43]. Importantly, this study links CD44 function and the alteration of EMT-related proteins with an increase of E-cadherin and a decrease of vimentin after the RNA interference-mediated reduction of CD44. EMT is characterized by the loss of epithelial characteristics including E-cadherin expression, and the gain of mesenchymal attributes such as vimentin signaling. It is a key phenomenon in wound healing, tissue repair and several diseases including cancers [44, 45]. During EMT, epithelial cells downregulate cell-cell adhesion systems, lose their polarity, and acquire a mesenchymal phenotype associated with an increased interaction with the extracellular matrix, as well as an enhanced migratory capacity [46]. Previously, CD44 has been reported to promote EMT through the activation of signaling by the Akt pathway in breast cancer [19]. Akt phosphorylation enhances cell migration and invasion in a variety of cancers [47, 48]. The functional mechanism can possibly be explained by the Akt signaling pathway reactivating NF-κB to induce the expression of Snail, and this in turn leads to the downregulation of E-cadherin expression [47]. Furthermore, type III intermediate filament vimentin is phosphorylated by Akt at Ser39, preventing its degradation and promoting cell migration [48]. Vimentin organization within the cell has important effects on the formation and function of invadopodia and lamellipodia during cellular invasion and migration [49]. In addition, GSK-3b degradation after PI3K/Akt activation leads to Slug overexpression followed by the induction of EMT [50]. We conclude that CD44-driving EMT processes *via* the Akt pathway plays a vital role in cancer metastasis in CCA by promoting cell migration.

Our work also shows that the depletion of CD44 affected cell invasion *via* the reduction of the matrix metalloproteinases (MMP)-9 signal, which is a family of zinc-dependent endopeptidases with important functions in extracellular matrix remodeling during development, and in inflammation and wound repair processes [51, 52]. Recently, MMP-9 has been shown to be required for invasion in breast cancer [53] and osteosarcomas [54]. The expression of MMP-9 is regulated by the Akt signaling pathway, and promotes cell invasion in many types of cancers, such as head and neck squamous cell carcinoma [55], gastric cancer [56] and renal cancer [57].

Based on these findings, the mechanism of action of the CD44 molecule involves the redox regulation contributing to CCA progression *via* activation of the ROS-Akt signaling pathway. In order to determine how CD44 is involved in redox regulation, we investigated the metabolic profile alteration of CCA cells with/without CD44 silencing using NMR-based metabolomics. The suppression of CD44 altered both the intra- and extra-cellular metabolic pathways, especially those involving metabolites related to glutathione synthesis and the TCA cycle. Their alteration was associated with various compounds including amino acids, glucose, and other intermediate metabolites. Previous reports showed that some cancers can escape from this mechanism *via* CD44v8-10 expression stabilizing xCT, thus reducing the ROS signal against cell death [12]. In addition, CD44 protein changed methionine-pool metabolites including spermidine and spermine, and reactive cysteine persulfides in human colon cancer cells, promoting cancer growth [58]. As it is widely known, glutamate and cysteine are the key amino acids involved in glutathione synthesis, and increasing levels of glutathione are one of the most important mechanisms for cancer drug resistance [59, 60]. Moreover, glutamine is one of the metabolites which is dysregulated in many cancers, and which is essential for cell proliferation through many pathways [61]. Importantly, it has been recently reported that the disruption of the glutamine metabolic pathways improves the efficacy of gemcitabine treatment *via* the alteration of protein glycosylation, expression and functional effects such as EGFR signaling, including downstream AKT-mTOR pathways, the MAPK pathway, as well as redox enzymes [62]. Furthermore, glutamine is hydrolyzed by glutaminases to become glutamate and inorganic ammonia [63]. Suppression of glutaminases has shown anti-tumor effects in several cancer models, and is currently under clinical investigation [64, 65]. Intracellular glutamine is commonly known as a key substrate required for cell growth and metabolism, as it serves as a carbon source to energize the TCA cycle, and transfers nitrogen for proteins, nucleotides and hexosamine synthesis [66–68]. A current report indicates that cisplatin-resistant cells show an increased consumption of glutamine [69]. In the current study, CD44 silencing promoted an accumulation of glutamine extracellularly, while intracellular glutamine was reduced. These circumstances might improve sensitivity to cancer treatment *via* regulation of the AKT-mTOR pathways, as well as redox homeostasis. In this study, we elucidated, for the first time, the metabolic profile changes after CD44 silencing which led to increased levels of ROS accompanied with decreased levels of essential compounds for glutathione synthesis, including cysteine and glutamate, as well as glutamine. This resulted in the activation of the Akt pathway. These events could promote CCA progression and metastasis.

## Conclusion

We present the first evidence that the molecular mechanisms of CCA proliferation, migration and invasion is regulated by the CD44-mediated Akt signaling pathway through metabolic profile alteration. Collectively, CD44 plays a crucial role in CCA progression, and therapy targeted to CD44-positive cells that can potentially improve the efficiency of CCA treatment.

## Supporting information

S1 Table The fold changes of intracellular metabolite profiling of CCA cells. (DOCX)

S2 Table The fold changes of extracellular metabolite profiling of CCA cells. (DOCX)

S1 Fig (TIF)
