# Supplementary material for: CD44 modulates metabolic pathways and altered ROS-mediated Akt signal promoting cholangiocarcinoma progression
Source: PLoS One. 2021 Mar 29;16(3):e0245871. doi: 10.1371/journal.pone.0245871 (PMC8007026; doi:10.1371/journal.pone.0245871)
Supplement: S1 Table — (DOCX) [file pone.0245871.s001.docx]

**S1 Table. The fold changes of intracellular metabolite profiling of CCA cells**

| **Metabolites** | **HMDB ID** | **KEGG ID** | **Log_2_ FC** | |
| --- | --- | --- | --- | --- |
|  |  |  | **CD44shRNA#1** | **CD44shRNA#2** |
| Acetate | HMDB0000042 | C00033 | -1.05 | -0.35 |
| 5-Hydroxytryptophan | HMDB0000472 | C01017 | -1.27 | -0.09 |
| 5-Methoxytryptophol | HMDB0001896 | - | -0.81 | -0.20 |
| Acetyl carnitine | HMDB0000201 | C02571 | 1.37 | -0.36 |
| Alanine | HMDB0000161 | C00041 | -0.88 | 0.06 |
| Alpha-aminoisobutyrate | HMDB0001906 | C03665 | -1.03 | -0.16 |
| Alpha-epsilon-diaminopimelate | HMDB0001370 | C00666 | -1.22 | -0.06 |
| Beta-hydroxybutyrate | HMDB0000357 | C01089 | -1.02 | -0.28 |
| Carnitine | HMDB0000062 | C00318 | -0.86 | -0.08 |
| Citrate | HMDB0000094 | C00158 | -1.12 | -0.59 |
| Creatine | HMDB0000064 | C00300 | -0.87 | -0.21 |
| Cysteate | HMDB0002757 | C00506 | -1.04 | -0.10 |
| Cysteine | HMDB0000574 | C00097 | -1.12 | -0.32 |
| Formate | HMDB0000142 | C00058 | -0.24 | -0.23 |
| Glutamate | HMDB0000148 | C00025 | -1.17 | -0.21 |
| Glutamine | HMDB0000641 | C00064 | -0.99 | -0.19 |
| Homoserine | HMDB0000719 | C00263 | -1.10 | -0.20 |
| Indole-3-lactate | HMDB0000671 | C02043 | -1.31 | -0.23 |
| Inosine | HMDB0000195 | C00294 | -0.47 | -0.58 |
| Isoleucine | HMDB0000172 | C00407 | -1.04 | -0.27 |
| Kynurenine | HMDB0000684 | C00328 | -0.61 | -0.93 |
| Lactate | HMDB0000190 | C00186 | 0.41 | 0.12 |
| L-Homocysteic acid | HMDB0002205 | C16511 | -1.09 | -0.18 |
| N-epsilon-methyllysine | HMDB0002038 | C02728 | -1.07 | -0.04 |
| o-Hydroxyphenylacetate | HMDB0000669 | C05852 | -1.12 | -0.13 |
| p-Hydroxybenzoate | HMDB0000500 | C00156 | -1.16 | 0.00 |
| Picolinate | HMDB0002243 | C10164 | -0.85 | -0.42 |
| Quinolinate | HMDB0000232 | C03722 | -0.70 | 0.19 |
| Succinate | HMDB0000254 | C00042 | -1.13 | -0.32 |
| Tryptamine | HMDB0000303 | C00398 | -3.69 | -5.00 |
| Tryptophan | HMDB0000929 | C00078 | -1.63 | -0.91 |
| Uracil | HMDB0000300 | C00106 | 0.28 | 1.32 |
| Valine | HMDB0000883 | C00183 | 1.27 | -0.21 |
