# Supplementary material for: CD44 modulates metabolic pathways and altered ROS-mediated Akt signal promoting cholangiocarcinoma progression
Source: PLoS One. 2021 Mar 29;16(3):e0245871. doi: 10.1371/journal.pone.0245871 (PMC8007026; doi:10.1371/journal.pone.0245871)
Supplement: S2 Table — (DOCX) [file pone.0245871.s002.docx]

**S2 Table. The fold changes of extracellular metabolite profiling of CCA cells**

| **Metabolites** | **HMDB ID** | **KEGG ID** | **Log_2_ FC** | |
| --- | --- | --- | --- | --- |
|  |  |  | **CD44shRNA#1** | **CD44shRNA#2** |
| Acetate | HMDB0000042 | C00033 | -0.05 | 0.02 |
| 5-Hydroxytryptamine | HMDB0000259 | C00780 | 0.02 | 0.03 |
| Alanine | HMDB0000161 | C00041 | 0.16 | 0.24 |
| Alpha-aminoisobutyrate | HMDB0001906 | C03665 | 0.09 | 0.01 |
| Choline | HMDB0000097 | C00114 | -0.13 | -0.26 |
| Carnitine | HMDB0000062 | C00318 | 0.16 | 0.89 |
| Cysteate | HMDB0002757 | C00506 | -0.44 | 0.12 |
| Cysteine | HMDB0000574 | C00097 | 0.09 | 0.00 |
| Formate | HMDB0000142 | C00058 | 0.19 | 0.53 |
| Glutamate | HMDB0000148 | C00025 | -0.07 | -0.16 |
| Glutamine | HMDB0000641 | C00064 | 0.21 | 0.05 |
| Homoserine | HMDB0000719 | C00263 | -0.13 | 0.02 |
| Isoleucine | HMDB0000172 | C00407 | -0.03 | -0.19 |
| Kynurenine | HMDB0000684 | C00328 | 0.03 | 0.00 |
| Lactate | HMDB0000190 | C00186 | 0.24 | 0.27 |
| Methionine | HMDB0000696 | C00073 | 0.07 | 0.01 |
| N-Acetylhistamine | HMDB0013253 | C05135 | 0.02 | 0.08 |
| Lysine | HMDB0000182 | C00047 | 0.12 | 0.07 |
| Pyruvate | HMDB0000243 | C00022 | -0.16 | -0.70 |
| Alpha-hydroxybutyrate | HMDB0000008 | C05984 | -0.10 | -0.24 |
| p-Hydroxypheny-lacetate | HMDB0000020 | C00642 | 0.05 | 0.04 |
| Threonine | HMDB0000303 | C00398 | -0.13 | -0.46 |
| Tryptamine | HMDB0000167 | C00188 | -0.06 | 0.59 |
| Glucose | HMDB0000122 | C00031 | -0.54 | -1.69 |
| Valine | HMDB0000883 | C00183 | 0.06 | 0.02 |
