# Supplementary figures and images for: CD44 modulates metabolic pathways and altered ROS-mediated Akt signal promoting cholangiocarcinoma progression
Source: PLoS One. 2021 Mar 29;16(3):e0245871. doi: 10.1371/journal.pone.0245871 (PMC8007026; doi:10.1371/journal.pone.0245871)

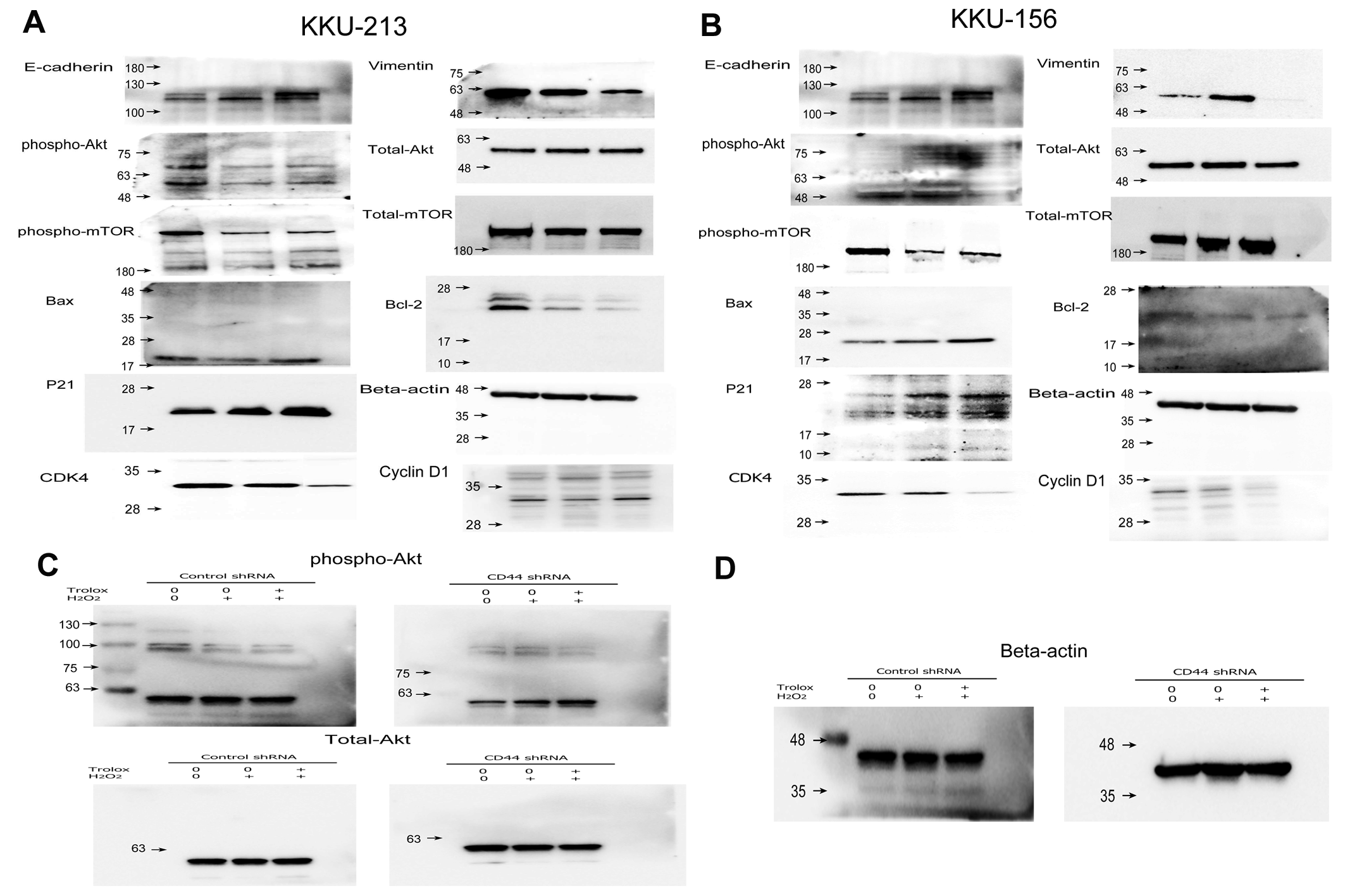

Supplement: S1 Fig — (TIF) [file pone.0245871.s003.tif]
